# Supplementary material for: Open versus Minimally Invasive Partial Nephrectomy: Trends and Outcomes from a Wide National Population-Based Database
Source: J Clin Med. 2024 Sep 14;13(18):5454. doi: 10.3390/jcm13185454 (PMC11431951; doi:10.3390/jcm13185454)
Supplement: Supplementary file 1 [file jcm-13-05454-s001.zip › jcm-3124986-supplementary.pdf]

Supplementary Table S1. International Classification of Diseases (ICD) 9 & 10 and Current Procedural Terminology (CPT) diagnosis and procedural codes utilized to identify renal masses and partial nephrectomy procedures.

	**ICD 9**	**ICD 10**	**CPT**
**Diagnosis**	Renal tumor: D-1890, D-23691	Renal tumor: D-C641, D-C642, D-C649, D-D300, D-3001, D-3002, D-C7A093, D3A093	
**Procedures**	Open Partial Nephrectomy:<br>Minimally invasive Partial Nephrectomy: P-554	Open Partial Nephrectomy: P-0TC00ZZ, P-0TC10ZZ, P-0TT00ZZ, P-0TT10ZZ, P-0TT20ZZ<br>Minimally invasive Partial Nephrectomy:<br>P-0TC04ZZ, P-0TC14ZZ, P-0TT04ZZ, P-0TT14ZZ, P-0TT24ZZ	Open Partial Nephrectomy: 50240, 50290<br>Minimally invasive Partial Nephrectomy: 50543, 50280

Supplementary Table S2. International Classification of Diseases (ICD) – 9 & 10 diagnosis codes utilized to identify socioeconomic determinants of health (SDOH)

	**ICD-9 Codes**	**ICD-10 Codes**
Education	V6129, 99550, 99551, 99553, 99554, 99559, V6121, 99555, V623	Z62898, Z629, Z620, Z6221, Z6222, Z6229, Z62810, Z62811, Z62812, Z62813, Z62819, Z62820, Z62821, Z62822, Z62890, Z62891, Z734, T7402XA, T7402XD, T7402XS, T7412XA, T7412XD, T7412XS, T7422XD, T7422XS, T7422XA, T7432XA, T7432XD, T7432XS, T7492XA, T7492XD, T7492XS, T7602XA, T7602XD, T7602XS, T7612XA, T7612XS, T7612XD, T7622XA, T7622XD, T7622XS, T7632XA, T7632XD, T7632XS, T7692XA, T7692XD, T7692XS, Z550, Z551, Z552, Z553, Z554, Z558, Z559, Z69010
Healthcare	V638, V639	Z749, Z748, Z753, Z754, Z758, Z759
Environmental	V8701, V8702, V8709, V8712, V8719, V1584, V1589, V8732, Z77021, Z77090, Z77098, Z77110, Z77111, Z77118, Z77122, Z77128, Z77123, Z77010, Z77012, Z77018, Z77121, V7181, 99580, 99581, 99582, 99583, 99584, 99585, E9600, E9601, E961, E9620, E9621, E9622, E9629, E963, E964, E9650, E9651, E9652, E9653, E9654, E9655, E9656, E9658, E9659, E966, E9680, E9681, E9682, E9683, E9684, E9685, E9686, E9687, E9688, E9689, E969, V8731, 9840, 9841, 9848, 9849, E8615, E8660, V1586, V1541, V1542, V1549, V6111, V6142	O9A311, O9A312, O9A313, O9A319, O9A32, O9A33, O9A411, O9A412, O9A413, O9A419, O9A42, O9A43, O9A511, O9A512, O9A513, O9A519, O9A52, O9A53, T7491XA, T7491XD, T7491XS, T7431XA, T7431XD, T7431XS, T7421XA, T7421XD, T7421XS, T7411XA, T7411XD, T7411XS, T7401XA, T7401XD, T7401XS, T7601XA, T7601XD, T7601XS, T7611XA, T7611XD, T7611XS, T7621XA, T7621XD, T7621XS, T7631XA, T7631XD, T7631XS, T7691XA, T7691XD, T7691XS, Z654, Z655, Z91410, Z91411, Z91412, Z91419, Z9142, Z9149, Z77011, Z77120, T560X1A, T560X1D, T560X1S, T560X2A, T560X2D, T560X2S, T560X3A, T560X3D, T560X3S, T560X4A, T560X4D, T560X4S, M1A10X0, M1A10X1, Z6911, Z6379
Social	V625, V603, V604, V6101, V6102, V6103, V6104, V6105, V6106, V6107, V6108, V6109, V613, V6141, V6149, V618, V619, V624, V6281, V6282, V6289, V629, V692, V693, V694, V698, V699, V695	Z603, Z605, Z608, Z609, Z600, Z602, Z604, Z650, Z651, Z652, Z653, Z658, Z659, Z6331, Z6332, Z634, Z635, Z636, Z6371, Z6372, Z6379, Z638, Z639, Z630, Z631, F439, Z732, Z733, Z7389, Z72810, Z72811, Z72820, Z72821, Z7289, Z73810, Z73811, Z73812, Z73819, Z739

Economic	V620, V621, V6229, V713, 99552, 99584, V602, V691, 9942, 9943, E9041, E9042, V600, V601, V6089, V609, V605	Z560, Z5689, Z569, Z563, Z562, Z561, Z564, Z565, Z566, Z5681, Z570, Z571, Z572, Z5731, Z5739, Z574, Z575, Z576, Z577, Z578, Z579, T730XXD, T730XXS, T730XXA, T731XXA, T731XXD, T731XXS, E630, E631, E638, E639, Z594, T738XXA, T738XXD, T738XXS, T739XXA, T739XXD, T739XXS, X58XXXA, X58XXXD, X58XXXS, Z724, E40, E41, E42, E43, E440, E441, E45, E46, E5111, E5112, E519, E52, E530, E531, E538, E539, E54, E550, E559, E560, E561, E568, E569, E58, E59, E60, E610, E611, E612, E613, E614, E615, E616, E617, E618, E619, E640, E641, E642, E643, E648, E649, Z590, Z591, Z595, Z596, Z597, Z598, Z599, Z91120

Supplementary Table S3. International Classification of Diseases (ICD) 9 & 10 diagnosis codes utilized to identify complications.

Complications	ICD 9	ICD 10
**Acute Kidney Injury**	5845, 5846, 5847, 5848, 5849	N170, N171, N172, N178, N179
**Blood transfusion**	990	Z513
**Deep vein thrombosis**	45340, 45342, 45341, 4532, 45382, 45384, 45385, 45386	I824Z1, I824Z2, I82220, I82621, I82622, I82623, I82629, I82A11, I82A12, I82A13, I82A19, I82B11, I82B12, I82B13, I82B19, I82C11, I82C12, I82C13, I82C19, I82401, I82402, I82403, I82409, I82411, I82412, I82413, I82419, I82421, I82422, I82423, I82429, I82431, I82432, I82433, I82439, I82441, I82442, I82443, I82449, I82451, I82452, I82453, I82459, I82461, I82462, I82463, I82469, I82491, I82492, I82493, I82499, I824Y1, I824Y2, I824Y3, I824Y9, I824Z3, I824Z9
**Disruption of surgical wound**	99830, 99831, 99832	T8130XA, T8131XA, T8132XA
**Ileus**	5601, 56081, 56089, 5609	K91
**Leakage**	56722, 56738, 56981	K632, K630, K651, K6811, K6819
**Pneumonia**	99731, 481, 4820, 4821, 48230, 48231, 48232, 48239, 48240, 48241, 48242, 48249, 48281, 48282, 48283, 48289, 4829, 4838, 485, 486, 4800, 4801, 4802, 4803, 4808, 4809	J120, J121, J122, J123, J1281, J1289, J129, J180, J181, J182, J188, J189, J13, J14, J150, J151, J1520, J15211, J15212, J1529, J153, J154, J155, J156, J157, J158, J159, J168
**Pulmonary embolism**	41511, 41512, 41519	I2601, I2609, I2690, I2693, I2699
**Sepsis**	99591, 99592, 99802, 0380, 03810, 03811, 03812, 03819, 0382, 0383, 03840, 03841, 03842, 03843, 03844, 03849, 0388, 0389	A327, A400, A401, A403, A408, A409, A4101, A4102, A411, A412, A413, A414, A4150, A4151, A4152, A4153, A4159, A4181, A4189, A419, R6520, R6521, T8144XA, T8144XD, T8144XS, T8112XA, T8112XD, T8112XS
**Urinary tract infection**	5990	N390
**Vascular injury**	9049, 9982	E3611, E3612, I978
